# Integratomics of Human Dermal Fibroblasts Treated with Low Molecular Weight Hyaluronic Acid

**DOI:** 10.3390/molecules26165096

**Published:** 2021-08-23

**Authors:** Silvia Radrezza, Gilda Aiello, Giovanna Baron, Giancarlo Aldini, Marina Carini, Alfonsina D’Amato

**Affiliations:** 1 Department of Pharmaceutical Sciences, University of Milan, Via L. Mangiagalli 25, 20133 Milan, Italy; silvia.radrezza@unimi.it (S.R.); gilda.aiello@unimi.it (G.A.); giovanna.baron@unimi.it (G.B.); giancarlo.aldini@unimi.it (G.A.); marina.carini@unimi.it (M.C.); 2 Department of Human Science and Quality of Life Promotion, Telematic University San Raffaele, 00166 Rome, Italy

**Keywords:** low-molecular weight hyaluronic acid, integratomics, lipidomics, proteomics, cosmetics

## Abstract

Hyaluronic acid (HA) is a glycosaminoglycan very common in commercial products from pharmaceuticals to cosmetics due to its widespread distribution in humans and its diversified physico-chemical proprieties. Despite its extended use and preliminary evidence showing even also opposite activities to the native form, the precise cellular effects of HA at low-molecular-weight (LWM-HA) are currently unclear. The ‘omics sciences currently in development offer a new and combined perspective on the cellular and organismal environment. This work aims to integrate lipidomics analyses to our previous quantitative proteomics one for a multi-omics vision of intra- and extra-cellular impact of different concentrations (0.125, 0.25, and 0.50%) of LMW-HA (20–50 kDa) on normal human dermal fibroblasts by LC-high resolution mass spectrometry (LC-HRMS). Untargeted lipidomics allowed us to identify 903 unique lipids mostly represented by triacylglycerols, ceramides, and phosphatidylcholines. According to proteomics analyses, LMW-HA 0.50% was the most effective concentration also in the lipidome rearrangement especially stimulating the synthesis of ceramides involved in skin hydration and reparation, cell signaling, and energy balance. Finally, integrative analyses showed 25 nodes covering several intra- and extra-cellular functions. The more complete comprehension of intra- and extra-cellular effects of LMW-HA here pointed out will be useful to further exploit its features and improve current formulations even though further studies on lipids biosynthesis and degradation are necessary.

## 1. Introduction

In recent decades, the use and commercial value of hyaluronic acid (HA), a glycosaminoglycan constitutively present at the extracellular matrix (ECM) level, is constantly increasing in the pharmaceutical, biomedical, and cosmetics industries thanks to its several biological functions. It shows an important role in cell signaling and proliferation, ECM structural organization, tissue reparation, angiogenesis, inflammatory, and immune response [1,2,3,4]. In the cosmetic field, it is widely used for anti-aging due to its enhancement of hydration, collagen stimulation, and tissue boost [5]. Nevertheless, it was demonstrated as HA’s biological functions and properties are strictly dependent on its molecular weight, also showing opposite effects between high-molecular-weight (HMW, if >10^6^ Da) and low-molecular-weight (LMW, if ≤10^6^ Da) HA [2,6]. In this regard, LMW-HA is recently becoming popular, especially in cosmetics and topical formulations, due to its easier skin penetration crossing *corneum stratum* and epidermis than HMW-HA, resulting in skin elasticity improvement, too [7,8,9]. 

Despite its increasing use, the molecular action of LMW-HA is still less known. In our previous work, we showed a significant impact of 20–50 kDa LWM-HA on the proteome profile of normal human dermal fibroblasts, especially at the highest concentration (0.50% LMW-HA) [10]. Here, applying high-resolution mass spectrometry and network analyses, we focused our attention on lipidome profile changes of human dermal fibroblasts (HDF) induced by the same treatment conditions (0.125, 0.25, and 0.50% LMW-HA, 24 h). The recent development of lipidomics techniques is clearly showing the importance and diversified roles of lipids in the intra- and extra-cellular functionality, including the skin layer. Indeed, lipids are far and away the only membrane raft structural components. Based on their physical and chemical properties, they are involved in many biological processes, including cell communication and differentiation, metabolism, energy balance, inflammatory and immune response [11,12]. Moreover, referring to the skin, it has been shown as an alteration of lipid composition and/or organization can influence the barrier proprieties implying several skin diseases such as atopic dermatitis or psoriasis [13]. So, having a lipids perspective is fundamental to provide a complete description of LMW-HA molecular effects.

Even if lipid analyses boosted by advanced mass spectrometry represent a strong point also for dermatological research [14], even more, is the integrative multiscale networking involving protein and lipid interactions. In this regard, our final goal was to integrate proteomics and lipidomics data offering a preliminary vision through large-scale systems biology approaches of LMW-HA effects at the cell level, which can support its use and pinpoint the potential benefits in dermocosmetics.

## 2. Results and Discussion

Label-free proteomics analyses conducted in our previous study showed a significant alteration of human dermal fibroblasts protein profile induced by LMW-HA, mainly at 0.50% of concentration (Table 1A) [10]. Indeed, proteins representing pathways such as cell proliferation and growth, extracellular matrix reorganization, proteoglycans biosynthesis, mitochondrial activity, cell adhesion, or wound healing were significantly upregulated. At the highest concentration (0.50%), LMW-HA also induced moderate upregulation of proteins involved in immune responses and inflammation processes however, without any impact on overall cells viability. Lipid metabolism-related proteins, such as SCP2, and HEXB, were also influenced by the treatment with 0.50% LMW-HA inducing an up-regulation.

Based on these results, here we extended our study to access the effect of LMW-HA on cellular lipidome with the aim to support multi-omics data integration.

### 2.1. Untargeted Lipidomics Profiling

Untargeted lipidomics analyses supported detection of 1380 lipids and the subsequent manual identification of 903 lipid molecular species (Table 1 and Table S1: Identified lipids), of which triacylglycerols (TGs), ceramides (Cer), and phosphatidylcholines (PCs) represented 45.2, 12.1 and 11.3% of identified lipidome, respectively.

**Figure 1:**
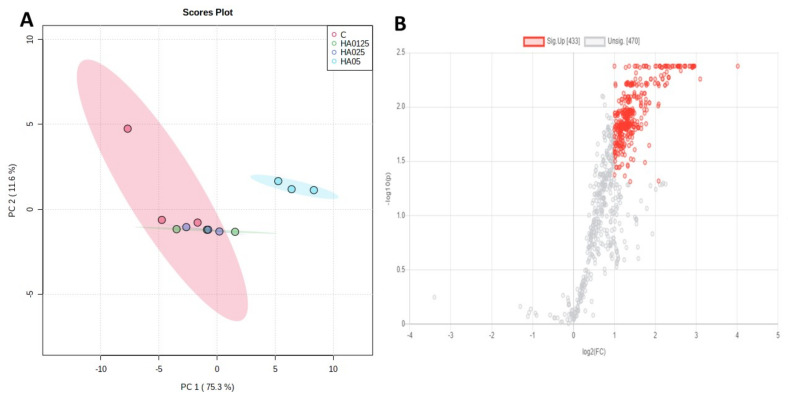
(**A**) Scoring plots reconstructed using PCA (PC1 vs. PC2). The red group corresponds to the control samples; green to those treated with 0.125% LMW-HA; violet to 0.25% LMW−HA and light blue to 0.50% LMW−HA treatment group; (**B**) Volcano Plot of LMW−HA 0.5% vs. C. In red the features significantly altered (fold change (FC) > 2, adjusted *p*-value < 0.05.

**Figure 2:**
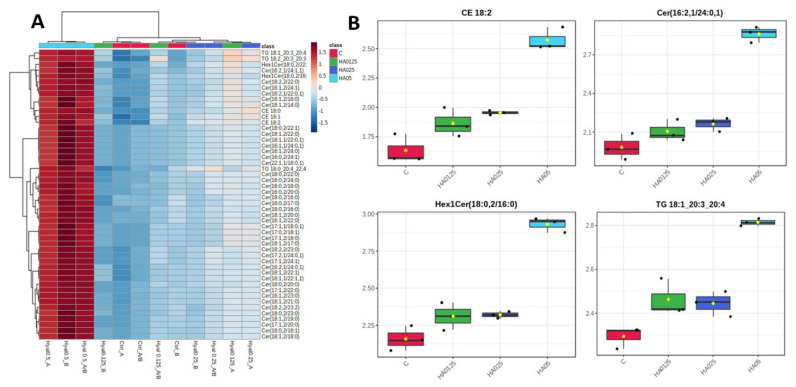
(**A**) Hierarchical clustering heatmaps of the 50 most significant altered lipids (one-way ANOVA and post-hoc analysis, *p* < 0.05) of all four groups. In red, the more expressed and related to the 0.50% LMW−HA group. Each colored cell on the map corresponds to a concentration value (in red those more expressed, in dark blue those with the lowest value) with samples in the rows and features in the columns; (**B**) box plot charts for representative altered lipids belonging to the main classes driving by one-way ANOVA (adjusted *p*-value (FDR) cut-off 0.05).

Analyzing the general lipidome profiles, 563 out of 903 features were significantly altered upon treatment with LMW-HA (one-way ANOVA, adjusted *p* value < 0.05, post-hoc analyses using Fisher’s LSD, Table S2: Significantly altered lipids). Moreover, principal component analysis (PCA) showed a clear cluster separation between cells treated with 0.50% LMW-HA and the remaining (controls, 0.125 and 0.25% LMW-HA groups), (Figure 1A). Insignificant differences instead between 0.125% LMW-HA vs controls and 0.25% LMW-HA vs controls based on the two-sample *t*-test and Wilcoxon rank sum test (*p* < 0.05) was observed, suggesting a significant cellular effect related only to the 0.50% LMW-HA as previously shown by the proteomics analyses (Table S2: Significantly altered lipids) [10]. In the box plot charts and hierarchical heatmap in Figure 2A,B, the definite 0.50% LMW-HA effect compared to the other groups on lipidome is well represented. Among the treatment conditions investigated, 0.50% LMW-HA led to significant changes in cells lipid composition, therefore, only these results are here discussed.

The 0.50% LMW-HA treatment induced, a significant alteration based on log2 FC (>1) of 433 features (out of 903, 47.95%; Figure 1B) belonging to cholesterol esters (CE; *n* = 4), ceramides (Cer; *n* = 105), hexosy-1-ceramides (Hex1Cer; *n* = 39), sphingomyelin (SM; *n* = 7), triacylglycerols (TG; *n* = 257) and diacylglycerols (DG; *n* = 13) vs controls. Similar increasing but not significant for 0.125% and 0.25% LMW-HA treatments. On the contrary, phospholipids classes i.e., phosphatidylcholines (PC), lyso-phosphatidylcholines (LPC), phosphatidylethanolamines (PE), lyso-phosphatidylethanolamines (LPE), phosphatidylinositols (PI), phosphatidylglycerols (PG) and phosphatidylserines (PS) were not statistically altered (*n* = 470, *p* > 0.05, FC < 2), except for LPC(18:0), LPE(18:0), PI(18:1_20:4) and five PS(PS 16:0_18:1, PS 18:0_18:1, PS 18:0_20:4, PS 18:0_22:6 and PS 18:1_18:1). 

Among those most differentially regulated by the treatment we found lipids belonging to cholesterol esters class such as CE (18:0) (log2 FC = 4.01, −log10(p) = 2.38) (Figure 1B) or CE (18:2) (log2 FC = 3.09, log10(p) = 2.26) (Figure 2B). Indeed, the major hyaluronan receptor, i.e., CD44, has demonstrated to induce keratinocyte differentiation and synthesis of cholesterol, precursor of CEs [15].

Further than CEs, there was an increasing of several ceramides and Hex1Cer including Cer(16:1, 2/23:0) (log2 FC = 2.96, log10(p) = 2.37) (Figure 2B), Cer(18:1, 2/21:0) (log2 FC = 2.94, log10(p) = 2.37), Hex1Cer(18:0, 2/22:0) (log2 FC = 2.73, log10(p) = 2.37) (Figure 2B) or Hex1Cer(18:0, 2/16:0) (log2 FC = 2.55, log10 (p) = 2.37).

Among the lipid classes in the skin, sphingolipids and ceramides are among the most important functional molecules at the stratum corneum and deeper fibroblasts layer, and they play a crucial role in the formation and maintenance of the skin barrier integrity [16,17,18]. Moreover, ceramides are necessary to link corneocytes into a waterproof barrier and enhance skin hydration that has an impact on cell morphology at the surface and deeper layers of the epidermis.

Sphingolipid composition can influence the identity, transition path and, lipid metabolic pathway in the establishment of wound repair of fibroblasts [18]. Nevertheless, being their activity depended on cell type and belonged subclasses, sphingolipids were further classified according to their structure, obtaining 43 (41%) non-hydroxy-sphingosine-ceramides, 26 (25%) sphingadienine-ceramides, 19 (18%) dihydro-ceramides, 10 (10%) phyto-ceramides, 6 (6%) deoxy-ceramides, and one (1%) dihydro-deoxy-ceramides, in line with what previously demonstrated [16,19].

Further than sphingolipids, 0.50% LMW-HA also induced an increase in TGs expression. The metabolism of TGs was observed to be influential in epidermal differentiation and in the skin’s barrier function, such as permeability [20]. Despite their role in softness and skin’s barrier functionality, the general increase of ceramides and triacylglycerols could also suggest deposition of lipid droplets that, at a certain level, can induce detrimental effects. Focused research on lipid droplets’ accumulation levels is therefore needed in the future.

### 2.2. Network Analyses Based on Integratomics

Although separated proteomics and lipidomics analyses allowed us to understand the cellular LWM-HA effects, integrating them in a multi-omics description provided an even further comprehension about its impact. To do that, we matched the significantly altered proteins (*n* = 495) and lipids by 0.50% LMW-HA treatment using ingenuity pathways analysis software (IPA). In total, 344 of 433 significantly altered lipids in the 0.50% LMW-HA group vs control were associated to the corresponding ID, needed for the integration, consulting genomic and molecular database such as KEGG, HMDB, PubChem, or ChEBI.

Twenty-five networks including 26 lipids belonging to different classes were found covering several intra- and extra-cellular functions, including lipids, vitamins and mineral metabolisms, cell signaling, and molecular transport (Table S3: Integratomics).

**Figure 3:**
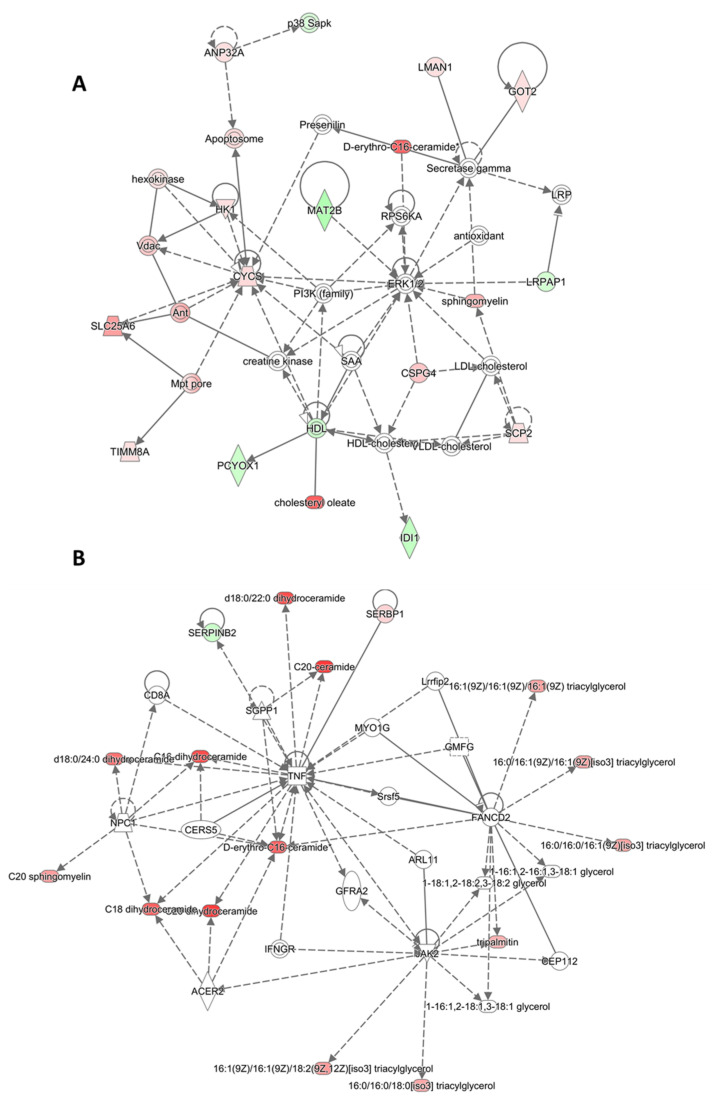
Networking between significantly altered proteins and lipids by LMW-HA 0.50% related to (**A**) mitochondrial activity and (**B**) inflammation. In red are the increased features covered by our input database, and in green are those decreased. The color intensity is positively related to the up or down-regulation.

Among all, integratomics analyses sustained a noticeable mitochondrial activity with LW-HA 0.50%, in line with the previous proteomics analyses [10]. Indeed, as showed in Figure 3A, we observed an up-regulation of respiration and energy providing proteins, including cytochrome C (CYCS) that plays an important role in cellular respiration, Aspartate aminotransferase (GOT2) implied in the metabolite exchange and in the long-chain free fatty acids, ADP/ATP translocase 3 (SLC25A6) required for the accumulation of coenzyme A in the mitochondrial matrix and mannose-specific lectin (LMAN1) involved in the sorting or recycling of proteins, lipids, or both. As involved lipids, we found cholesteryl oleate (CE (18:1(9Z))), d-erythro-C16-ceramide (Cer(d18:1/16:0)) and sphingomyelin (SM(d18:1/18:0)), all increased and with a role on cell signaling and differentiation, membrane stabilizer, energy storage and lipids transport. In addition, it was shown as ceramide synthesis is enhanced along with the rate of keratinocyte differentiation both in vitro and in vivo, supporting our hypothesis of induction of fibroblast maturation by the treatment [21]. Among their roles, intracellular ceramides act as the second messenger with pro-apoptotic functions in several tissues and cells as demonstrable by the modulation of related genes. Among them, we found MAT2B and CSPG4, both involved in cell proliferation pathways, CYCS also involved in cell death pathways, and apoptosome, a proteic complex needed for the apoptosis trigger (Figure 3A). Moreover, we saw a down-expression of SERPINB2, a negative regulator of apoptosis, related to the tumor necrosis factor (TNF) pathway, also involved in the process as an apoptotic enhancer **(**Figure 3B). In the skin, this activity could be explained as a self-renewal process applied to the regulation of keratinocyte proliferation/differentiation balance by exerting anti-proliferative and pro-apoptotic effects [21]. Apoptosis in human fibroblasts is also related to the contractility of the extracellular matrix [22]. In our case, the integratomics data also showed that LMW-HA 0.50% induced a fine, regulated apoptosis with a negative overall score associated with this pathway (z score = −2.23), supporting the hypothesis of controlled apoptosis.

Moreover, the moderate activation of TNF-mediated inflammation in response to the treatment previously showed by proteomics was also confirmed in lipidomics (Figure 3B), involving several lipids belonging to Cer, TGs, and SMs classes such as Cer(d18:0/18:0), TG(16:0/16:0/18:0) or SM(d18:1/20:0) (in the figure referred respectively as C18 dihydroceramide, 16:0/16:0/18:0[iso3] triacylglycerol and C20 sphingomyelin). In this case, ceramides and their derivatives have been largely investigated in the context of inflammation and immune response. Indeed, several inflammatory cytokines, including TNF-alpha, have been implicated in the regulation of ceramides production.

Hence, considering their diversified roles, the equilibrium between ceramide synthesis and degradation is essential for maintaining epidermal renewal and normal homeostasis. Indeed, an excessive accumulation may cause harmful inflammation and damages as UV-induced ones [23]. At the same time, upregulation of SERBP1 was observed (Figure 3B). SERBP1 can promote the resolution of inflammatory responses by inducing enzymes that synthesize suppressive unsaturated FAs supporting a good balance in the inflammation and the overall cell wellness [24,25].

Further than mitochondrial activity and moderate inflammation, integratomics showed a lipids involvement in networks (although less covered by our lipids and proteins identification) related to cell signaling, mobility, and transcription regulation (Network 18; Table S3: Integratomics; Figure S1), cells proliferation (Network 21; Table S3: Integratomics, Figure S2) and peroxisomal beta-oxidation pathway of fatty acids (Network 25; Table S3: Integratomics; Figure S3). In the first one (Network 18), as involved lipids, we found an up-regulation of Cer(d18:1/24:0), Cer(d18:1/22:0), and PC (16:0/22:0) all acting also in the cell signaling pathway. Moreover, as proteins related to lipids, we saw a down-regulation of PAFAH1B2 with a rule in lipid degradation, MESD that binds low-density lipoprotein receptors. Up-regulated proteins are also linked to cell reorganization, proliferation, and transcription, such as DYNLT1, active in the actin cytoskeleton regulation, and ANP32P, while multifunctional proteins are also involved in cell proliferation, cell cycle progression, and transcription. As acting lipids in Network 21, we found CE(18:0), Cer(d18:1/18:0), and LysoPC(18:0/0:0) all up-regulated and mainly involved in energy storage, membrane stabilization, and cell signaling processes. As proteins related to lipid processing, we saw a down-regulation of IAH1, operating in lipid degradation. In the end, despite having sparse connections in Network 25, we found an up-regulation of PC(14:0/20:4), PC(16:0/18:3), PC(18:2/20:4), SM(d18:1/22:0), and Cer(d18:1/23:0) acting in the network centered on PPARG that controls the peroxisomal beta-oxidation pathway of fatty acids and is a key regulator of adipocite differentiation and glucose homeostasis. Up-regulation also of MGLL, a protein related to the conversion of monoacylglycerides to free fatty acids and glycerol.

## 3. Materials and Methods

### 3.1. Chemicals

Primary cell line (NHDF-Ad 28887), glutamine and penicillin-streptomycin antibiotic were purchased by Lonza Bioscence (Basel, Switzerland); Dulbecco’s Modified Eagle’s Medium (DMEM), trypsin-EDTA 0.5% 10×, sodium piruvate were obtained by Gibco^®^ (Thermo Fisher Scientific, Bremen, Germany); Renovyhal 20–50 kDa here referred as low-molecular hyaluronic acid (LMW-HA) was purchased by Soliance (Pomacle, France); Fetal bovine serum (FBS), and phosphate buffered saline (PBS) were purchased by Euroclone^®^ (Milano, Italia); methanol (MeOH), acetonitrile (MeCN), 2-propanol (i-PrOH), and formic acid (FA) (all ULC/MS-CC/SFC grade) were purchased from Biosolve (Valkenswaard, Netherlands); Methyl-tert-butyl-ether (≥99%, MTBE), ammonium formate (NH_4_HCO_2_) MS grade was purchased from Sigma-Aldrich (Taufkirchen, Germany); SPLASH^®^ LIPIDOMIX^®^ Mass Spec Standard was purchased by Avanti Polar Lipids Inc. (Alabaster, AL, USA). Water was purified in-house (resistance > 18 MΩ cm^−1^; total organic content < 10 ppb) on a PureLab Ultra Analytic System (ELGA Lab Water, Celle, Germany).

### 3.2. Cell Culture and Treatment

The adult normal human dermal fibroblasts (NHDF-Ad 28887) were cultured as a monolayer in DMEM containing 10% FBS, 1% glutamine, and 1% penicillin-streptomycin antibiotic, at 37 °C in a humidified atmosphere of 5% CO_2_ as previously described [10]. Three experimental conditions were planned considering the results of cell viability assays and the usual concentrations in the cosmetic products. NHDF-Ad (7th passage) seeded in T75 flasks were treated in biological duplicate with 0.125, 0.25, and 0.50% LMW-HA (*w*/*v*, in milliQ-H_2_O) respectively for 24 h considering the physiological turn-over. Untreated cells were used as control. The viability of cells was evaluated using MTT reduction assay (Sigma-Aldrich) and Real-Time Glo-MT kit assay (Promega, Madison, WI, USA) as previously described [10].

### 3.3. Sample Preparation

Upon 24 h treatment, cells were trypsinized (3 mL of 0.05% trypsin-EDTA, *v*/*v* in PBS), transferred into tubes, counted using the automatic counter TC20™ (Bio-Rad^®^), pelleted in cold PBS by two cycles of centrifuging (400× *g*, 4 °C for 5 min) and the supernatant removed. 500 μL of antioxidant solution (0.1% *w*/*v* BHT in water) was used for the resuspension and the solution moved into a new tube followed by centrifugation (10 min, 4 °C, 1000× *g*) and discharge of the supernatant. The volume of solution used for the final resuspension was adjusted based on the cell count and 50 μL corresponding to around 1.50 × 10^5^ cells taken for lipid extraction.

### 3.4. Lipid Extraction

Splash^®^ Lipidomix^®^ (5 μL) was added to 50 μL of cell suspension in 0.1% BHT in water, left on ice for 15 min, and lipids were extracted using the MTBE protocol [26]. All solvents contained BHT (0.1% *w*/*v*) and were cooled on ice before use. Briefly, 375 μL of MeOH were added to each sample and vortexed for 5 s. Then, 1250 μL of MTBE was added, followed by 5 s vortex and incubation (1 h, 4 °C, 210 rpm). The phase separation was induced by adding 315 μL of H_2_O, vortex for 5 s and 10 min of incubation (4 °C, 210 rpm). Once centrifugated (4 °C, 10 min, 2000× *g*), the upper phase was collected into a new tube, dried under vacuum (Eppendorf concentrator 5301, 1 mbar). Before the LC-MS analyses, lipids extracts were dissolved in 100 μL i-PrOH and vortexed. Total quality control samples (QCs, *n* = 4) were obtained mixing 10 μL each, group pool samples (*n* = 4, i.e., Ctrl A_B, LMW-HA 0.125% A_B, LMW-HA 0.25% A_B, LMW-HA 0.50% A_B) mixing 10 μL of 2 replicates for a total of 16 samples.

### 3.5. Mass Spectrometry

Lipid were separated using reverse phase chromatography on a Vanquish Horizon system (Thermo Fisher Scientific, Bremen, Germany) equipped with an Accucore C18 column (150 × 2.1 mm; 2.6 μm, 150 Å; Thermo Fisher Scientific, Bremen, Germany). Gradient elution with solvent A (MeCN/H_2_O, 1:1, *v*/*v*) and B (i-PrOH /MeCN/H_2_O, 85:10:5, *v*/*v*), both containing 5 mM ammonium formate (NH_4_HCO_2_) and 0.1% FA (*v*/*v*) was used. Separation was performed at 50 °C with a flow rate of 0.3 mL/min using the following gradient: 0–20 min 10–86% B; 20.1–22 min 86–95% B; 22.1–26 min 95% B; 26.1–34 min 10% B. Mass spectrometry detection was performed with a Q-Exactive Plus Hybrid Quadrupole-Orbitrap mass spectrometer (Thermo Fisher Scientific, Bremen, Germany) equipped with a HESI probe. Mass spectra were acquired in positive and negative mode with the following source parameters: sheath gas, 40 L/min; auxiliary gas, 10 L/min; sweep gas, 1 L/min; spray voltage 3.5 kV (−2.5 kV); spray current, 10 μA; capillary temperature, 300 °C; S-lens RF level, 35 and aux gas heater temperature, 370 °C.

Full MS spectra were acquired at a resolution setting for *m*/*z* 200 at 140,000, scan range 380–1200 *m*/*z* (negative), and 250–1200 *m*/*z* (positive), automatic gain control (AGC) target 1e6 counts, maximum injection time (IT) 100 ms. MS/MS spectra were acquired applying a data-dependent acquisition (DDA, top 15) method was used at a resolution of 17,500 at *m*/*z* 200, AGC target of 2e5, and a maximum IT of 60 ms. An isolation window for precursor selection was 1.2 *m*/*z* and a stepped collision energy (CE 10-20-30 eV) was used for HCD.

### 3.6. Data Analysis

Lipids identification strategy was based on merged results obtained by LipidHunter 2 RC_3 (https://github.com/SysMedOs/lipidhunter) [27], LipoStar (version 1.3.2 × 64, Molecular Discovery, Hertfordshire, UK) [28] and MSDial (http://prime.psc.riken.jp/compms/msdial/main.html) [29] followed by manual annotation in Skyline v. 20.2.0.343. Relative quantification was based on the determination of area under the curve (AUC) for each lipid correctly identified then normalized by AUC of the used ISTD to the corresponding lipid species and original the cell number. Then, MetaboAnalyst v 5.0 online software (https://www.metaboanalyst.ca/) was used to perform statistical analyses (*p*-value < 0.05, fold change > 2, FDR) [30].

Finally, integratomics analyses between lipidomics and previously obtained proteomics data [10] were done through Ingenuity Pathways Analysis software (IPA, Qiagen, last release). Briefly, each of the significantly altered lipids was assigned an ID corresponded to KEGG (Kyoto Encyclopedia of Genes and Genomes), HMDB (Human Metabolome Database), PubChem, or ChEBI (Chemical Entities of Biological Interest) database. Based on the ID frequency matches, we selected HMDB as the source for the integration. Protein’s gene name and related log2 ratio were then included for the final input database (See Table S3: Integratomics).

## 4. Conclusions

To conclude, this study highlights as both proteome and lipidome of normal human dermal fibroblasts are influenced by low-molecular-weight hyaluronic acid, in particular at 0.50% of concentration. Moreover, to our knowledge, this is the first study that describes LMW-HA in vitro effects combining proteomics and lipidomics analyses in a multi-omics approach. The previous proteomics results [10] were not only confirmed but also corroborated by lipidomics and integratomics results. Indeed, mitochondria functionality, cells maturation, and lipids metabolism were demonstrated as well. About lipidome changes, we saw a particular increase of ceramides, hex-ceramides, and cholesterol esters involved in the skin moisturizing and epidermis renewal, supporting the beneficial role of low-molecular-weight as a cosmetic ingredient. Nevertheless, the correct balance between their synthesis and metabolism is essential for skin wellness, and further studies aim to verify the lipids droplets hypothesis are necessary.

## Figures and Tables

**Table 1:** (**A**) Summary of detected, filtered, and significantly altered features in lipidomics and proteomics analyses [10]; * unique by structure, ** Volcano plot; FC > 2, *p* value < 0.05; (**B**) Total identified lipids at bulk and structure level divided by classes; CE = cholesterol esters, Cer = ceramides, Hex1- Hex2Cer = 1-2-hexosylceramides, SM = sphingomyelin, TG = triacylglycerols, DG = diacylglycerols, PC = phosphatidylcholines, LPC = lyso-phosphatidylcholines, PE = phosphatidylethanolamines; LPE = lyso-phosphatidylethanolamines; PI = phosphatidylinositols; PG = phosphatidylglycerol; PS = phosphatidylserine.

A			
	Conditions	Lipidomics	Proteomics
Identified features *		903 (694 in Pos)	2328
Significantly altered **	0.50% LMW-HA vs. C	433	495
	0.25% LMW-HA vs. C	/	149
	0.125% LMW-HA vs. C	/	39
**B**			
**Class**	**Bulk (n)**	**Unique by Structure (n)**	**%**
CE	27	27	3.0
Cer	54	109	12.1
Hex1Cer	21	56	6.2
Hex2Cer	6	12	1.3
SM	26	26	2.9
TG	116	408	45.2
DG	31	56	6.2
PC	40	102	11.3
LPC	3	3	0.3
PE	32	75	8.3
LPE	4	4	0.4
PI	11	12	1.3
PG	3	3	0.3
PS	10	10	1.1
Tot	384	903	100

## Data Availability

The data presented in this study are available in Supplementary Materials.

## Supplementary Materials

The following are available online. Figure S1: Network 18. Figure S2: Network 21. Figure S3: Network 25. Table S1: Identified lipids; Table S2: Significantly altered lipids; Table S3: Integratomics.

## Sample Availability

Samples of the compounds are not available from the authors.

## Abbreviations

HA, hyaluronic acid; LMW-HA, low-molecular-weight hyaluronic acid; HMW-HA, high-molecular-weight hyaluronic acid; LC-HRMS, liquid chromatography-high resolution mass spectrometry; HDF, human dermal fibroblasts; DDA, data-dependent acquisition; NHDF, normal human dermal fibroblasts; CE, cholesterol esters; Cer, ceramides; Hex 1-Hex2Cer, 1-2-hexosylceramides; SM, sphingomyelin; TG, triacylglycerols; DG, diacylglycerols; PC, phosphatidylcholines; LPC, lyso-phosphatidylcholines; PE, phosphatidylethanolamines; LPE, lyso-phosphatidylethanolamines; PI, phosphatidylinositols; PG, phosphatidylglycerol; PS, phosphatidylserine.
