# Supplementary figures and images for: Integratomics of Human Dermal Fibroblasts Treated with Low Molecular Weight Hyaluronic Acid
Source: Molecules. 2021 Aug 23;26(16):5096. doi: 10.3390/molecules26165096 (PMC8399884; doi:10.3390/molecules26165096)

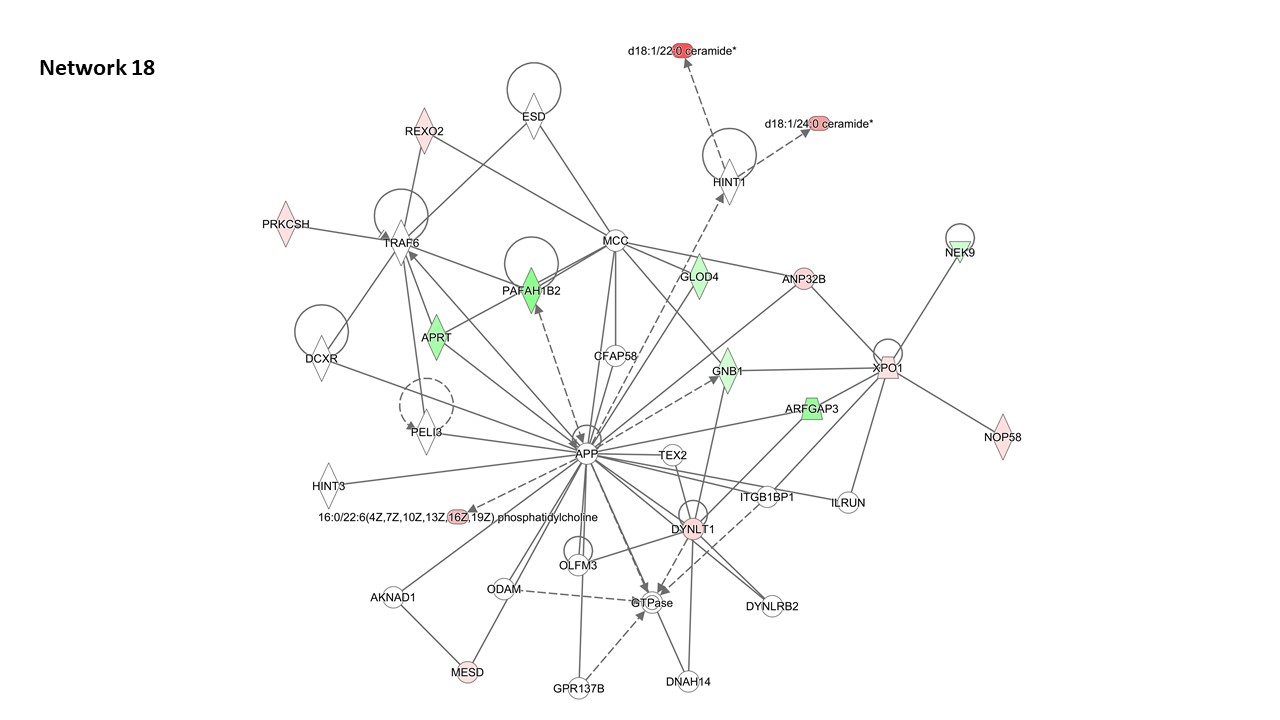

Supplement: Supplementary file 1 [file molecules-26-05096-s001.zip › molecules-1334966-supplementary/Revision_supplementaryTables and Figures/Suppl Fig 1.jfif]

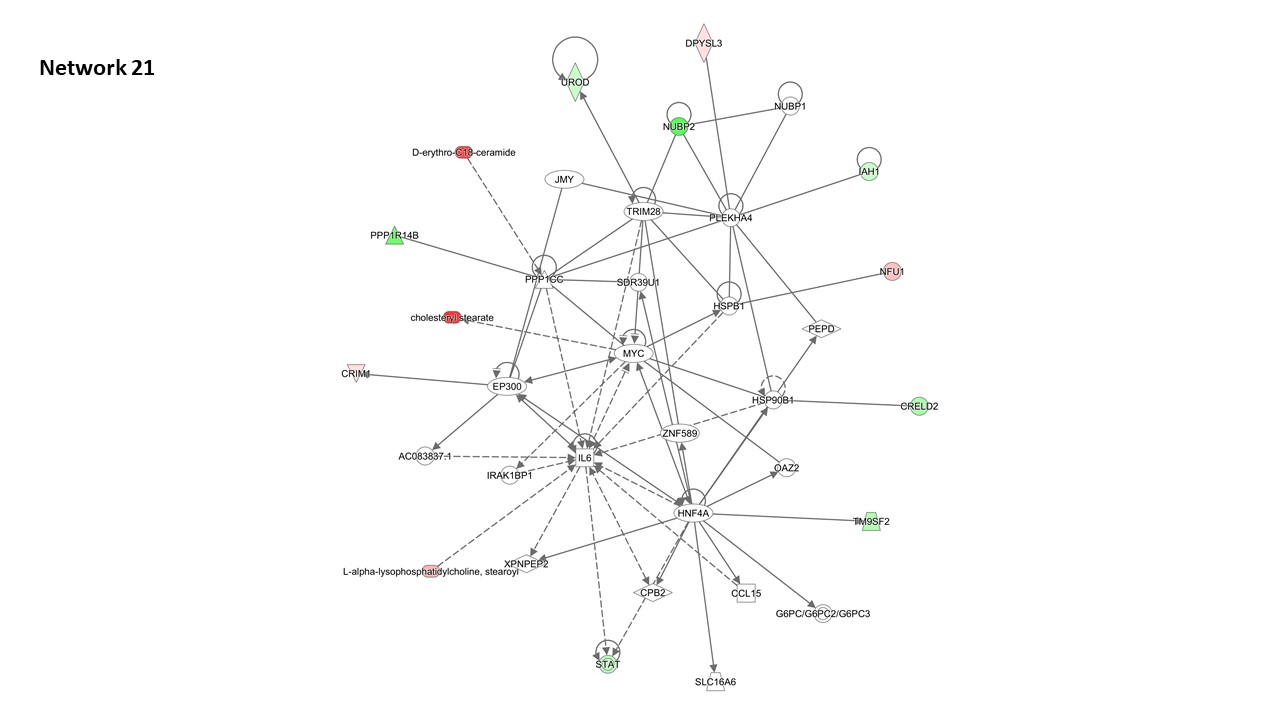

Supplement: Supplementary file 1 [file molecules-26-05096-s001.zip › molecules-1334966-supplementary/Revision_supplementaryTables and Figures/Suppl Fig 2.jfif]

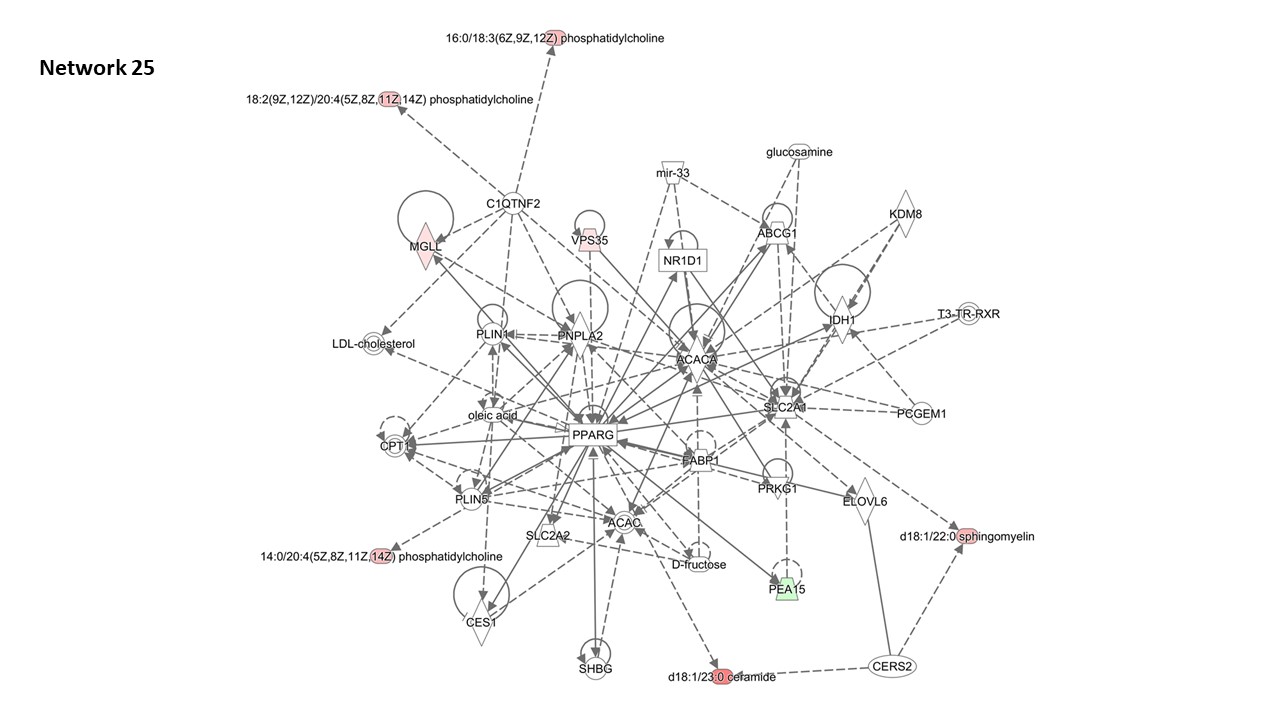

Supplement: Supplementary file 1 [file molecules-26-05096-s001.zip › molecules-1334966-supplementary/Revision_supplementaryTables and Figures/Suppl Fig 3.jfif]
